# Supplementary material for: Characterization of Host-Associated Microbiota and Isolation of Antagonistic Bacteria from Greater Amberjack (Seriola dumerili, Risso, 1810) Larvae
Source: Microorganisms. 2023 Jul 26;11(8):1889. doi: 10.3390/microorganisms11081889 (PMC10456766; doi:10.3390/microorganisms11081889)
Supplement: Supplementary file 1 [file microorganisms-11-01889-s001.zip › microorganisms-2473609-supplementary.pdf]

Characterization of host-associated microbiota and isolation of antagonistic bacteria from greater amberjack (*Seriola dumerili*, Risso, 1810) larvae

Vasiliki Paralika [1], Fotini Kokou [2], Stelios Karapanagiotis[3] and Pavlos Makridis [1*]

[1] Department of Biology, University of Patras, 26504 Rio, Greece; bio3235@upnet.gr
[2] Aquaculture and Fisheries Group, Department of Animal Sciences, Wageningen University, 6700 AH Wageningen, The Netherlands; fotinikokou@wur.nl
[3] Galaxidi Marine Farm S.A., 33200 Galaxidi, Greece, karapanagiotis@galaxidimarine.farm

* Authors to whom correspondence should be addressed: makridis@upatras.gr, Tel.: +30-2610-969224

Suppementary material

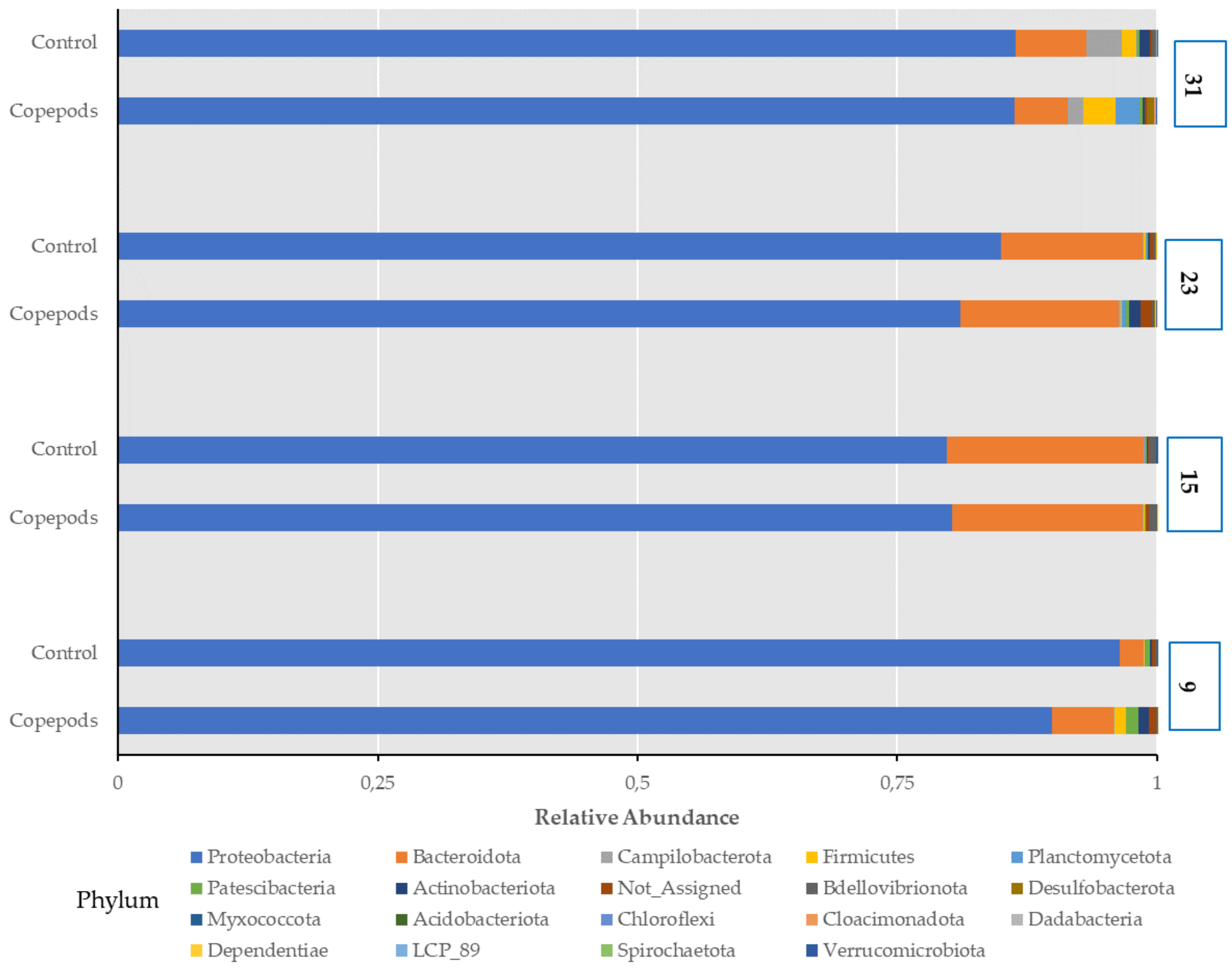

Figure S1. Relative abundance of bacteria phyla in all sampling points (control, copepods; diet) (9, 15, 23, 31; dah).

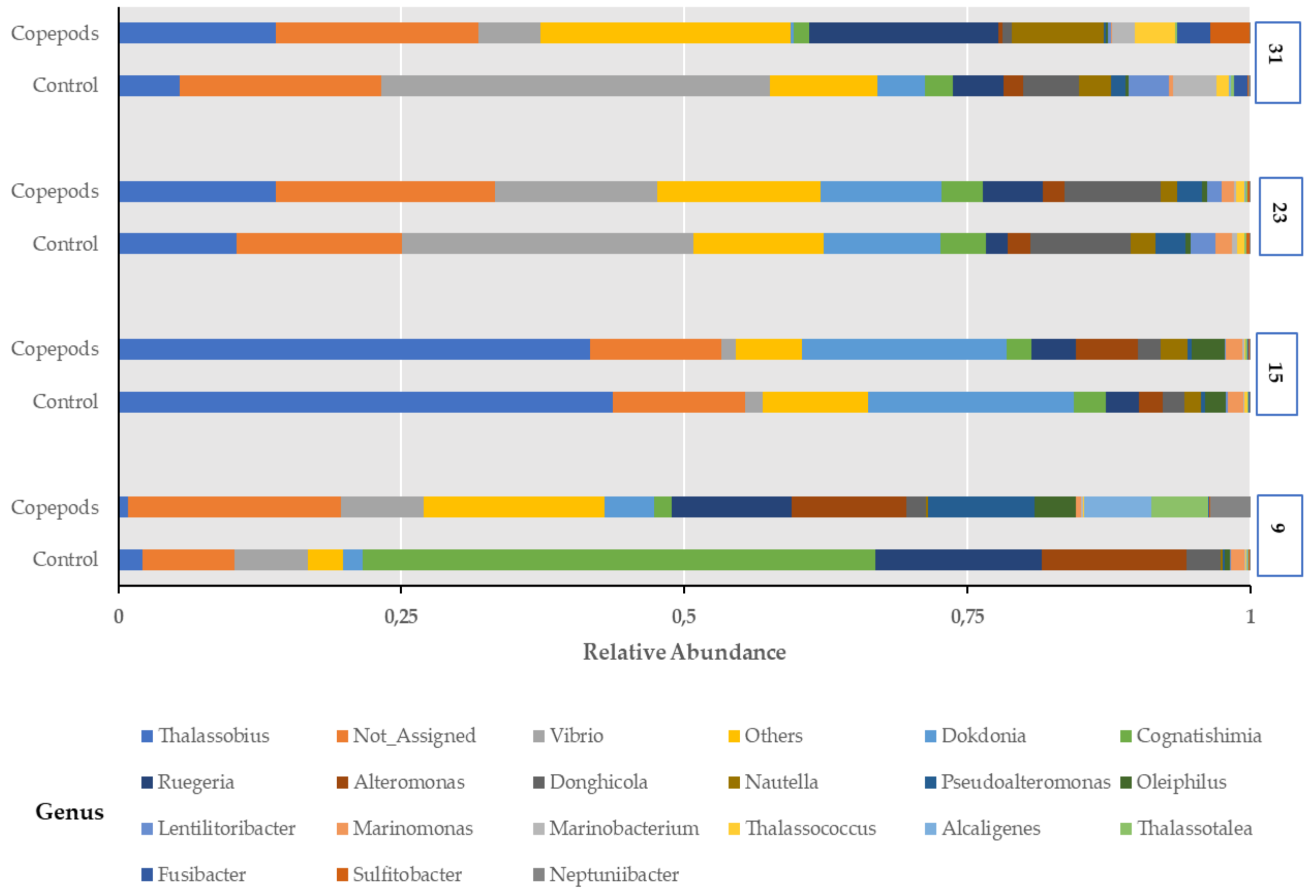

Figure S2. Relative abundance of prokaryotic genera in all sampling points (control, copepods; diet) (9, 15, 23, 31; dah).

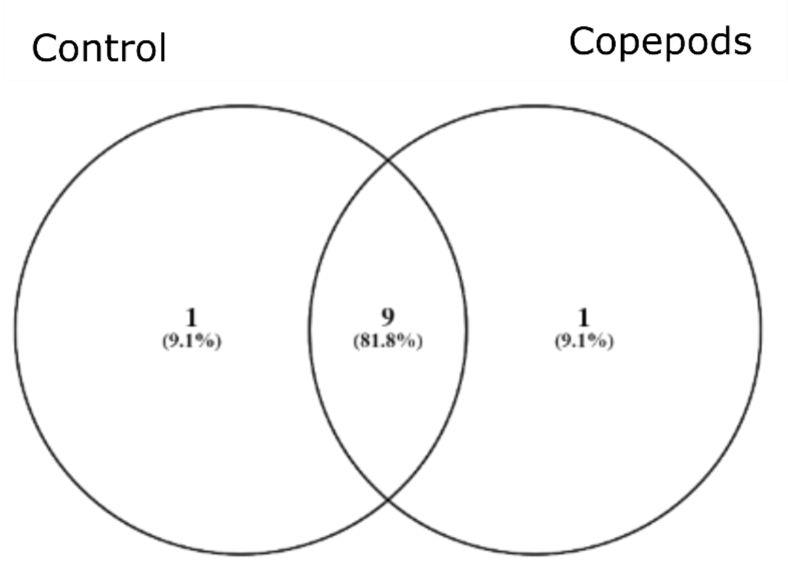

Figure S3. Core shared and unique genera of *S.dumerili* larvae fed with the control and the copepods feeding protocols for all the experimental period. Core ASVs, defined as genera present, with a prevalence of 50% and occurring at 1% minimum of relative abundance.

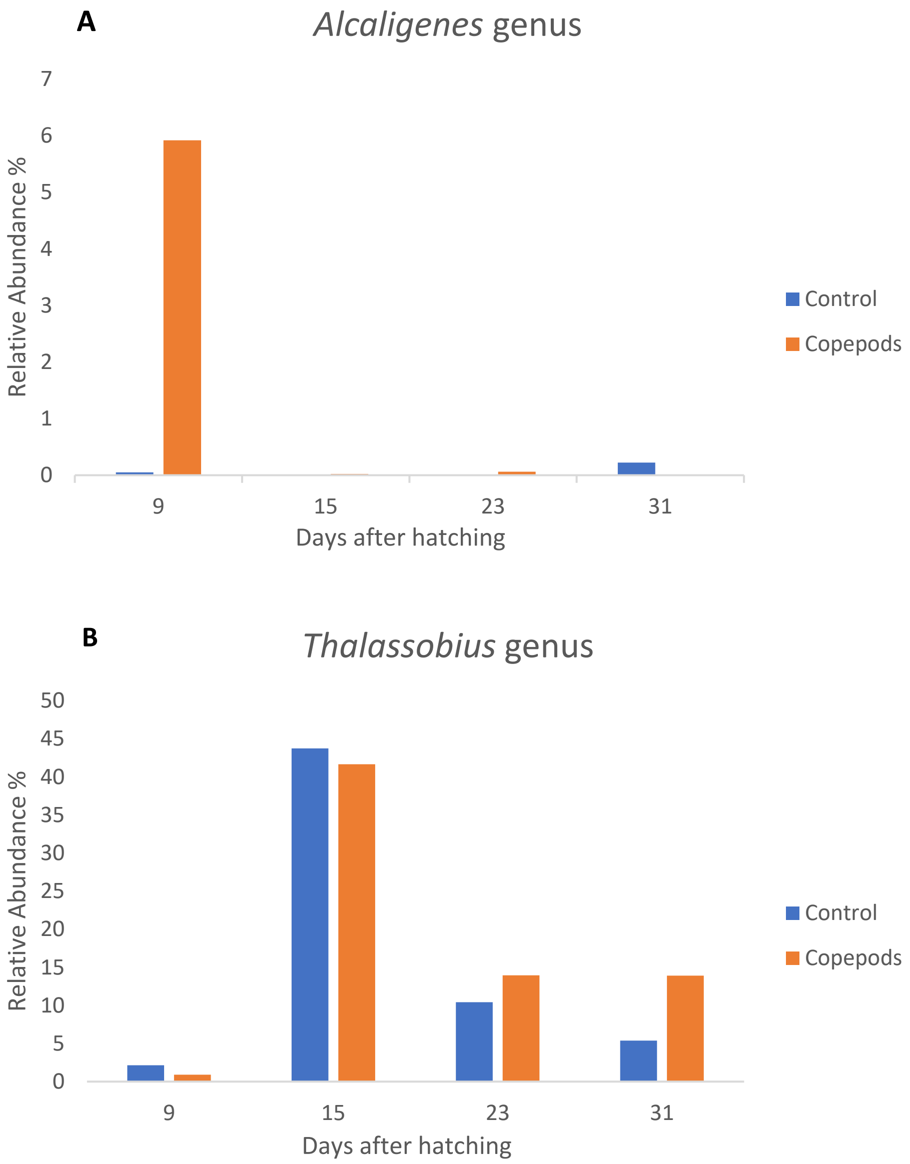

Figure S4. Relative abundance of the significantly ($p<0.05$) **A.** differential abundant *Alcaligenes* genus and **B.** most abundant *Thalassobius* genus, between the control and the copepod feeding protocols 9, 15, 23, 31 dah.
